# Late Rickets

**Published:** 1905-04-22

**Authors:** 


### LATE RICKETS.

The name "late rickets" has been applied to certain rare cases of rachitic lesions in young adults, these being not infrequently individuals who during infancy had shown no evidence of the ordinary form of the disease. Dr. R. W. Marsden[1] has recently published a case believed to be of this order, the details of which are as follows.

The patient was a girl of 18¾, of healthy stock, the father and mother being both in good health. The available history showed no taint of syphilis in the fa[illegible]ily. The girl in question had shown no definit[illegible] evidence of rickets during childhood, though [illegible] is mentioned that she did not walk until she was two years old. She was always of small stature for her age, but seemed in every way normal, in that she took part in the common tasks and games of childhood. The one peculiarity about her was a somewhat freakish appetite; that is to say, she ate readily of meat, but evinced a great distaste for vegetables. Also she was a voracious eater of salt, and lost no opportunity of getting it. Six months before she came under notice she began to feel great weariness upon exertion, with loss of appetite and of muscular power. Two months later she complained of pains in the legs on standing or walking, and presently developed enlargements of her knees and ankles, accompanied by knock-knee. When seen she was quite unable to walk.

On examination she proved to be very diminutive, with a degree of development more suited to a girl of 10 or 11, with the exception that her breasts were well formed. Her face was sallow, and the sclerotics, where exposed, were pigmented. Her skin was pigmented and brown at the root of the neck, in the axillæ, and on the front and sides of the abdomen. Her teeth were irregular and overcrowded. The epiphyses of her long bones were much enlarged, and the shafts short, while the bones were the seat of patchy tenderness.

Some doubt still lingers round the existence of late rickets. Emmett Holt, with his wide experience, has never seen it. But several cases have been reported in this country, and of one of them which came to the dead house, Barlow and Abercrombie reported that "the bones present all the features of the infantile disease." Comby regards these cases, as did Trousseau, as identical with osteomalakia, but this identity has been disproved by Kassowitz.

Dr. Marsden is satisfied that the case was not a case of Addison's disease complicated by osteomalakia, though on the facts presented it would seem hard definitely to dismiss the former possibility. He discusses the points upon which a diagnosis of rickets may be justly founded. Clutton is of opinion that the epiphysial enlargement is the critical evidence of rickets. The author lays more stress upon the depth of the layer of epiphysial cartilage as shown by the $x$-rays, and quotes some data showing the excessive thickness of this layer in rickets. For instance, in a normal child of three years it was ·4 mm., while in a rickety child of two-and-a-half it was 2·2 mm.

Dr. Marsden holds that the disease is not of late origin, strictly speaking, but is the outcome of a developmental anomaly dating back to the early years of youth, though for a varying number of years quiescent, and accepts Macewen's dictum that knock-knees, bow-legs, and the femoral and tibial curves of adolescence all alike originate in rickets.

[1] Edin. Med. Jour., April 1905.

